# Feasibility and implementation of a daily safety brief at a children's hospital-in-a-hospital

**DOI:** 10.3389/fped.2026.1752719

**Published:** 2026-05-25

**Authors:** Weijen William Chang, Patricia Fontaine, Stephanie Adam, Joanna Beachy, Mark Heelon, Christine McKiernan, Deborah Naglieri-Prescod, Kristy Parker, Robert Rothstein, Charlotte Boney

**Affiliations:** 1 Department of Pediatrics, UMass Chan School of Medicine Baystate, Springfield, MA, United States; 2 Department of Pediatrics, University of Central Florida, Orlando, FL, United States; 3 Department of Pediatrics, Nemours Children's Hospital Florida, Orlando, FL, United States; 4 Department of Pediatrics, Baystate Medical Center/Baystate Children’s Hospital, Springfield, MA, United States

**Keywords:** children's hospital, daily safety brief, feasibility, patient safety, quality assessement, situational awareness

## Abstract

**Background:**

The daily safety brief (DSB) is a structured approach to enhancing patient safety and readiness, widely used in free-standing children's hospitals. This observational study examines the implementation and feasibility of a DSB within a children's hospital embedded in an adult healthcare system—a unique challenge requiring adaptation to an infrastructure primarily designed for adult care.

**Methods:**

Using a descriptive, observational design, we tracked safety concerns reported during DSBs over a 12-month period across inpatient units and the pediatric emergency department. Safety concerns were categorized using a predefined taxonomy and reviewed by the implementation team.

**Results:**

The implementation process confirmed the feasibility of integrating pediatric safety efforts within an adult system. While qualitative feedback suggested improved communication and situational awareness, this study did not measure direct improvements in patient safety outcomes, and the single-center design limits generalizability. The reduction in reported safety concerns over time should be interpreted cautiously, as changes in reporting may reflect cultural or behavioral factors rather than true safety improvements.

**Conclusions:**

This initiative highlights the potential for embedding pediatric safety practices within broader hospital operations, warranting further investigation using controlled designs with objective patient safety outcome measures.

## Introduction

Implementing daily safety briefs (DSB) has improved patient safety and operational readiness in free-standing children's hospitals (1, 2), reduced invasive device use in pediatric intensive care units (3), and increased situational awareness in pediatric oncology units (4). Implementing DSBs within a children's hospital-in-a-hospital, where pediatric units are embedded within a general hospital, remains underexplored. Prior literature has described DSBs as focused, time-limited meetings designed for patient safety and daily readiness (5), in which all departments involved in delivering care to pediatric patients address immediate safety issues, review recent safety events, anticipate potential risks, and provide status updates that could impact safety or operations through either in-person or remote participation (6, 7).

Recent literature has further examined the impact and conceptual underpinnings of safety huddles. A systematic review by Franklin et al. (8) synthesized 24 studies and found generally positive results for unit-based huddle programs, though the prevailing study design was uncontrolled pre-post comparison, and the authors noted that high-quality evidence regarding hospital-wide huddles remains in its earliest stages. Pimentel et al. (9) conducted a scoping review of huddles at the frontlines of clinical care, finding that huddles are increasingly prevalent across diverse health care settings and are generally aimed at improving team communication and coordination, though knowledge of their implementation and effectiveness remains fragmented. The Huddle Up for Safer Healthcare (HUSH) project evaluated patient safety huddle implementation across 92 wards in five UK NHS hospitals and found that while huddles were embedded in 64 wards, mean fidelity scores were modest, highlighting the challenge of maintaining implementation quality at scale (10). Rotteau et al. (11) explored physician engagement through Medical Safety Huddles across six sites in Toronto, Canada, finding that physician-led huddles enhanced engagement with organizational quality and safety efforts.

International studies have further expanded the evidence base. Lee et al. (12) conducted a mixed-methods study of a 12-week safety huddle intervention in surgical units at a tertiary teaching hospital in Seoul, Korea, finding significant improvements in six of nine measured outcomes, including organizational learning, situation monitoring, and speaking-up climate. Similarly, Moraes et al. (13) evaluated patient safety culture before and after the implementation of a safety huddle at a municipal hospital in Ceará, Brazil, using a quasi-experimental design and demonstrating statistically significant improvements in patient safety perception and adverse event reporting. A study by Chen et al. (14) at Taichung Veterans General Hospital in Taiwan also showed that huddle interventions positively impacted patient safety culture among medical team members. These international studies highlight the adaptability of huddle-based interventions across diverse cultural and healthcare contexts, while also revealing that most research remains limited by single-center designs and a reliance on self-reported outcomes.

Together, these studies underscore both the promise and the methodological limitations of the existing evidence base. The present study contributes to this literature by describing the implementation of a DSB in a children's hospital-in-a-hospital—a model that has not been previously examined—and discusses the unique contextual factors that differentiate this setting from free-standing pediatric facilities.

## Methods

This initiative aimed to implement a 15-minute DSB at a children's hospital-in-a-hospital and prospectively track daily metrics related to departments reporting safety concerns, types of safety concerns, and the number of safety concerns over time. The study employed a descriptive, observational design without a comparison group or pre-implementation baseline period. Safety concerns were categorized using a predefined taxonomy developed by the implementation team, with categories reviewed iteratively during the initial months of deployment to ensure consistency. It should be noted that this study did not include pre-post statistical analysis or a control group, which limits causal inference.

The impetus for the DSB implementation arose after completing the Solutions for Patient Safety (SPS) Culture Wave, which highlighted a gap in structured safety communications. Solutions for Patient Safety is a collaborative network of children's hospitals in the United States and Canada focusing on safety culture, leadership engagement, and data-driven learning (15). The implementation process involved several key steps:

1. **Sponsor/Stakeholder Identification**: Two senior physicians and nursing executive sponsors were identified, and stakeholders were organized to spearhead the initiative.
2. **Training/Development**: Key stakeholders and personnel attended SPS leadership methods training.
3. **Focus Group Formation**: A focus group was established to develop the structure, organize department training sessions, and ensure adherence to the new protocols.
4. **Communication/Branding**: Invitations were extended to relevant departments and units. A logo was created for branding purposes, and a structured template was designed to guide the meetings.
5. **Template Design**: Critical components to review by each department were determined, including but not limited to:
  - Staffing concerns
  - Unit status
  - Employee safety concerns or issues
  - Environmental safety issues
  - Patient safety concerns
  - Family concerns
  - “Watcher” patients
  - Drug shortages

No protected health information was recorded in this template.

The DSB was rolled out on October 4, 2021, with all departments affecting the Children's Hospital participating daily from 11:00–11:15 a.m. Minutes of reported safety issues were recorded in a cloud-based document. Issues needing further follow-up were recorded and re-visited at the end of subsequent DSBs until resolved or “rolled up” to higher-level huddles.

Daily minutes were reviewed to quantify the safety issues in each category. Safety concerns were categorized by the DSB facilitator using a predefined taxonomy that included staffing, medical equipment/devices, medication/fluids, provision of care, environmental safety, patient identification/documentation, and other categories. Categories were established *a priori* based on existing safety event classification frameworks and refined during the first month of implementation. To promote coding reliability, the facilitator and a second member of the implementation team independently reviewed a subset of reported concerns during the first three months to ensure consistent categorization. Discrepancies were resolved through discussion. Descriptive statistics were used to summarize the frequency and distribution of safety concerns over time. No inferential statistical analyses were performed, as the study was designed as a feasibility and implementation review rather than an outcomes study.

This study was a descriptive review of an ongoing quality improvement initiative and did not involve direct interaction with or intervention on human subjects. No protected health information was collected or recorded. The study was reviewed by the Department of Pediatrics at UMass Chan School of Medicine Baystate, and the department chair determined that it constituted a review of an operational quality improvement program and was therefore exempt from Institutional Review Board (IRB) approval. As such, written informed consent from participants was not required.

## Results

**Figure 1:**
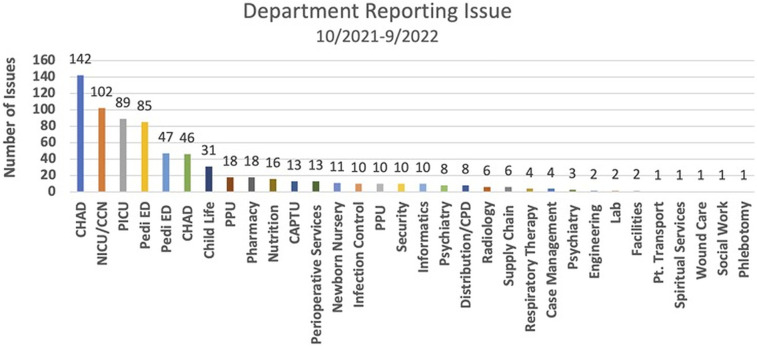
Safety issues reported by departments during DSB from October 2021 to September 2022. The bar graph illustrates the number of safety issues reported across various hospital departments over a 12-month period. The Children & Adolescents (Acute Care) Unit (CHAD) reported the highest number of issues (142), followed by the Neonatal Intensive Care Unit/Continuing Care Nursery (NICU/CCN, 102) and the Pediatric Intensive Care Unit (PICU, 89). Other departments reported varying numbers of safety concerns, with the lowest numbers observed in Phlebotomy, Social Work, and Spiritual Services (each reporting one issue). CHAD, children & adolescents (Acute Care) unit; PPU, pediatric procedure unit; CCN, continuing care unit; CAPTU, child/adolescent psychiatric treatment unit.

**Figure 2:**
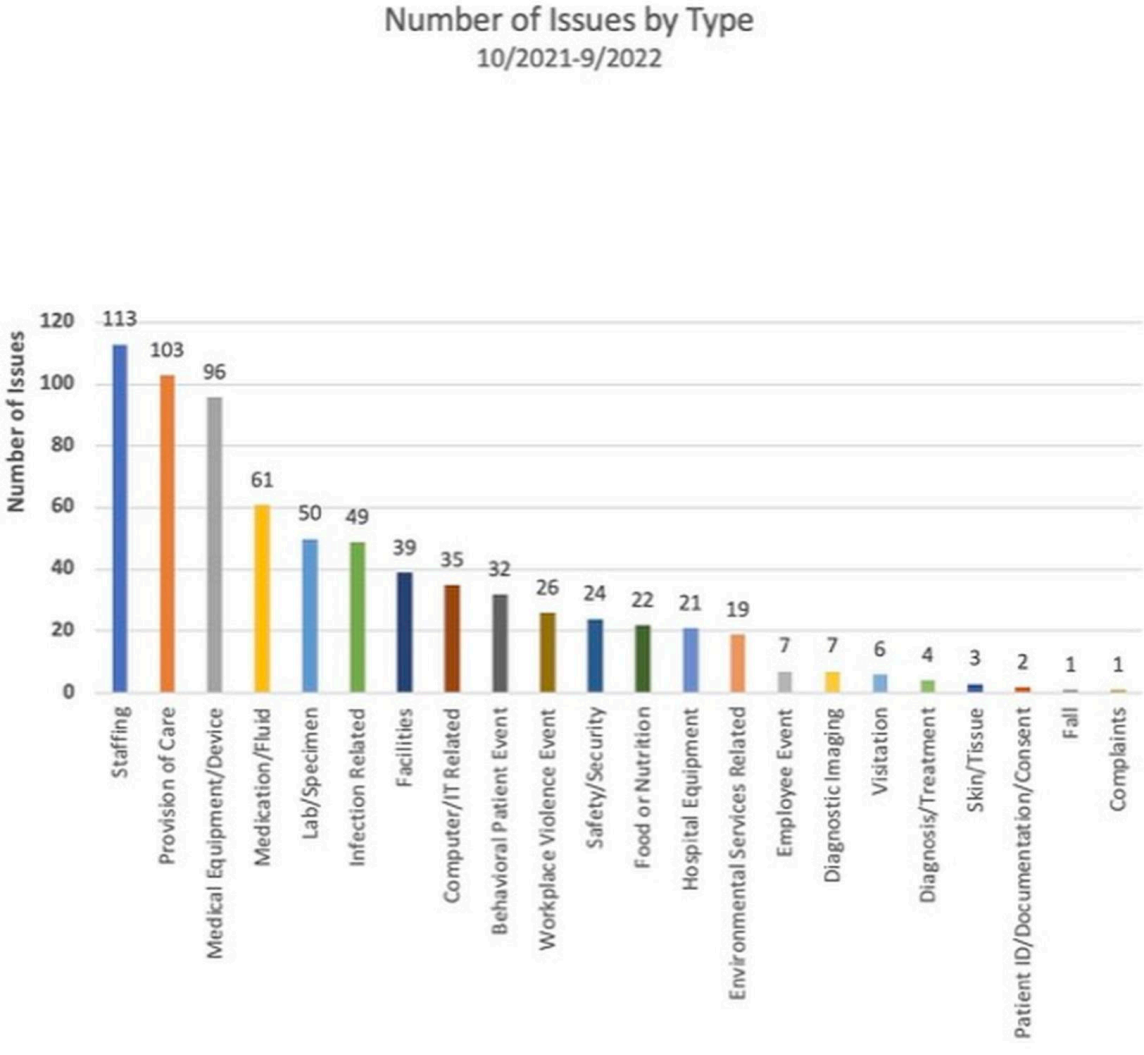
Number of safety issues raised during DSB by category, October 2021—September 2022. This bar graph presents the distribution of safety issues reported across different categories during the designated 12-month period. The most frequently reported issue was staffing concerns (113 reports), followed by provision of care (103) and medical equipment/device issues (96). Other notable categories include medication/fluid (61), lab/specimen-related issues (50), and infection-related concerns (49). Less frequent reports involved diagnosis/treatment, patient identification/documentation/consent, falls, and complaints, each with fewer than five reports. This categorization highlights the key areas of concern related to patient safety within the reporting period.

During the initial four months, most safety concerns originated from inpatient units (acute care unit, NICU/CCN, PICU) and the pediatric emergency department, which persisted over the initial 12-month period (Figure 1). Concerns were predominantly related to medical equipment/devices, medication/fluids, and the provision of care. After 12 months, top concerns transitioned to staffing, provision of care, and medical equipment/devices. (Figure 2)

Quantitatively, reported safety concerns in the DSB decreased 72% from a 7-day average of 6.0 concerns/day initially to 1.7 concerns/day after four months. Subsequently, there was an increase in daily concerns raised. Overall, the mean reported safety concerns was 3.4 per day (range: 0–12). The average daily census for inpatient units remained relatively stable over the 12-month period (monthly patient-days ranged from 1,383 to 1,695; mean 1,496 patient-days/month). However, this reduction should be interpreted with caution. The decline in reported concerns may reflect changes in reporting behavior, increased efficiency in issue resolution, normalization of the reporting process, or cultural shifts rather than a true reduction in safety hazards. Without a comparison group or pre-implementation baseline data, it is not possible to attribute the observed changes solely to the DSB intervention. No inferential statistical tests were applied to assess the significance of these trends. Although this observational study was not designed to measure changes in patient safety outcomes, serious safety events (SSE) and serious harm events (SHE) did not increase during the study period (4 SSEs total; SHE range 1–5/month against a hospital centerline of 2.0), providing reassurance that the implementation occurred against a stable safety backdrop.

Qualitative feedback was collected informally from DSB participants over the study period through verbal comments during and after DSB sessions, as well as written feedback solicited via email and departmental meetings. Feedback was reviewed by the implementation team and organized thematically to identify recurring patterns. While this feedback was not subjected to formal qualitative analysis (e.g., thematic coding with inter-rater reliability), participant comments consistently reflected increased situational awareness, enhanced focus on safety, and improved triaging of safety issues. Representative comments included:

- “[This] helped the teams get to know each other better”
- “Our chair feels this is a much better, much safer hospital than just 2 years ago and most definitely 5 years ago.”
- “[We are] able to detect issues/events more effectively because we are actively asking, ‘are there issues?’”
- “Transparency increased/guilt and fear have decreased–culture is more of a sense we want to hear about events.”
- “Shared commitment–daily safety brief has been a vehicle to move things faster.”

## Conclusions

Implementing the DSB within a children's hospital-in-a-hospital setting required deliberate engagement of stakeholders and adaptation of existing models, but proved feasible and anecdotally beneficial despite many reporting departments not being exclusively pediatric-focused. Challenges included maintaining time constraints of DSB meetings and ensuring that discussions remained focused, with more extensive issues redirected to appropriate venues. While the DSB appeared to enhance communication and situational awareness based on participant feedback, several important limitations must be acknowledged.

First, this study did not measure the effect on direct patient safety outcomes such as adverse event rates, hospital-acquired conditions, or patient harm metrics. The reliance on reported safety concerns as a surrogate metric is a significant limitation, as changes in reporting frequency may reflect cultural shifts or reporting fatigue rather than genuine safety improvements. Second, the qualitative feedback, while encouraging, was collected informally and was not subjected to rigorous qualitative analytic methods (e.g., systematic thematic analysis with coding frameworks and inter-rater reliability assessment). This limits the strength of conclusions drawn from participant perceptions. Third, the absence of a comparison group or pre-implementation baseline data precludes causal inference and statistical comparison. Fourth, the single-center design limits the generalizability of findings to other children's hospital-in-a-hospital settings, which may differ in organizational structure, safety culture, and resource availability.

The sustainability of the DSB also warrants consideration. While this initiative was maintained over a 12-month period, long-term adherence to daily safety briefs may be influenced by leadership turnover, competing operational priorities, and participant fatigue. Future studies should incorporate structured sustainability assessments and examine potential confounders, such as concurrent quality improvement initiatives, seasonal variations in patient volume, and staffing changes that may independently affect safety outcomes.

Despite these limitations, the successful implementation of a DSB in a children's hospital-in-a-hospital represents a meaningful contribution to the literature, as this model has not been previously described. Our findings align with the broader evidence base suggesting that safety huddles improve team communication and situational awareness (8, 9, 16–18), while extending this work to a unique institutional context. Unlike free-standing children's hospitals where DSBs have been most commonly studied (1, 2), the hospital-in-a-hospital model requires coordination across departments that serve both adult and pediatric populations, presenting distinct logistical and cultural challenges. International studies from Korea (12), Brazil (13), and Taiwan (14) have similarly demonstrated the adaptability of huddle interventions across diverse healthcare contexts, though direct comparisons are limited by differences in study design, setting, and outcome measurement.

## Data Availability

The data analyzed in this study is subject to the following licenses/restrictions: Datasets can be provided upon request. Requests to access these datasets should be directed to weijenwchang@gmail.com.

## References

[1] Saysana M McCaskey M Cox E Thompson R Tuttle LK Haut PR. A step toward high reliability: implementation of a daily safety brief in a Children's Hospital. J Patient Saf. (2017) 13(3):149–52. 10.1097/PTS.0000000000000131 25119785

[2] Sediva I Snelling L. Daily safety briefs (DSB) focus on improving safety at hasbro Children's Hospital. R I Med J (2013). (2018) 101(2):23. 29490319

[3] Tarrago R Nowak JE Leonard CS Payne NR. Reductions in invasive device use and care costs after institution of a daily safety checklist in a pediatric critical care unit. Jt Comm J Qual Patient Saf. (2014) 40(6):270–8. 10.1016/s1553-7250(14)40036-9 25016675

[4] Chapman LR Molloy L Wright F Oswald C Adnum K O'Brien TA Implementation of situational awareness in the pediatric oncology setting. Does a ‘huddle’ work and is it sustainable? J Pediatr Nurs. (2020) 50:75–80. 10.1016/j.pedn.2019.10.016 31770680

[5] Hatva E. Daily briefing promotes hospital-wide transparency and patient safety. Biomed Instrum Technol. (2013) 47(6):489–92. 10.2345/0899-8205-47.6.489 24328970

[6] Donnelly LF Basta KC Dykes AM Zhang W Shook JE. The daily operational brief: fostering daily readiness, care coordination, and problem-solving accountability in a large pediatric health care system. Jt Comm J Qual Patient Saf. (2018) 44(1):43–51. 10.1016/j.jcjq.2017.04.010 29290246

[7] Institute for Healthcare Improvement. Safety briefings (2004). Available online at: https://www.ihi.org/sites/default/files/SafetyBriefings.pdf (Accessed 2004).

[8] Franklin BJ Gandhi TK Bates DW Huancahuari N Morris CA Pearson M Impact of multidisciplinary team huddles on patient safety: a systematic review and proposed taxonomy. BMJ Qual Saf. (2020) 29(10):844–53. 10.1136/bmjqs-2019-009911 32265256

[9] Pimentel CB Snow AL Carnes SL Shah NR Loup JR Vallejo-Luces TM Huddles and their effectiveness at the frontlines of clinical care: a scoping review. J Gen Intern Med. (2021) 36(9):2772–83. 10.1007/s11606-021-06632-9 33559062 PMC8390736

[10] Stapley E Sharples E Lachman P Lakhanpaul M Wolpert M Deighton J. Fidelity and the impact of patient safety huddles on teamwork and safety culture: an evaluation of the huddle up for safer healthcare (HUSH) project. BMC Health Serv Res. (2021) 21:1038. 10.1186/s12913-021-07080-1 34598704 PMC8487146

[11] Rotteau L Othman D Dunbar-Yaffe R Fortin C Go K Mayo A Physician engagement in organisational patient safety through the implementation of a medical safety huddle initiative: a qualitative study. BMJ Qual Saf. (2023) 33(1):33–42. 10.1136/bmjqs-2022-015725 37468150

[12] Lee SE Dahinten VS Kim E Lee SH Han SY Kim PJ A safety huddle intervention in in-patient surgical units: a mixed-methods study. J Nurs Manag. (2023) 2023:8929993. 10.1155/2023/8929993 40225626 PMC11919141

[13] Moraes MVA Almeida ILS Carvalho REFL. Patient safety culture assessment before and after safety huddle implementation. Rev Esc Enferm USP. (2024) 57:e20230270. 10.1590/1980-220X-REEUSP-2023-0270en 38358114 PMC10868519

[14] Chen WC Wu MJ Chen HH Lin SP Wu CY Chin CS Impacts of huddle intervention on the patient safety culture of medical team members in medical ward: one-group pretest-posttest design. J Multidiscip Healthc. (2023) 16:3599–607. 10.2147/JMDH.S434185 38024136 PMC10680486

[15] Lyren A Coffey M Shepherd M Lashutka N Muething S, Group SPSL. We will not compete on safety: how Children's Hospitals have Come together to hasten harm reduction. Jt Comm J Qual Patient Saf. (2018) 44(7):377–88. 10.1016/j.jcjq.2018.04.005 30008350

[16] Aldawood F Kazzaz Y AlShehri A Alali H Al-Surimi K. Enhancing teamwork communication and patient safety responsiveness in a paediatric intensive care unit using the daily safety huddle tool. BMJ Open Qual. (2020) 9(1):e000753. 10.1136/bmjoq-2019-000753 32098776 PMC7047506

[17] Goldenhar LM Brady PW Sutcliffe KM Muething SE. Huddling for high reliability and situation awareness. BMJ Qual Saf. (2013) 22(11):899–906. 10.1136/bmjqs-2012-001467 23744537 PMC6288816

[18] Rowan BL, Anjara S, De Brún A, MacDonald S, Kearns EC, Marnane M, et al. The impact of huddles on a multidisciplinary healthcare teams' Work engagement, teamwork and job satisfaction: a systematic review. J Eval Clin Pract. (2022) 28(3):382–93. 10.1111/jep.13648 35174941

